# Static and dynamic posture control in postlingual cochlear implanted patients: effects of dual-tasking, visual and auditory inputs suppression

**DOI:** 10.3389/fnint.2013.00111

**Published:** 2014-01-16

**Authors:** Laurence Bernard-Demanze, Jacques Léonard, Michel Dumitrescu, Renaud Meller, Jacques Magnan, Michel Lacour

**Affiliations:** ^1^Integrative and Adaptive Neurosciences Laboratory, UMR 7260 CNRS/Aix-Marseille UniversityMarseille, France; ^2^Service d'ORL et Chirurgie de la Face et du Cou, Assistance Publique Hopitaux de marseille, CHU NordMarseille, France

**Keywords:** posture control, cochlear implanted patients, dual-tasking, visual input, auditory input

## Abstract

Posture control is based on central integration of multisensory inputs, and on internal representation of body orientation in space. This multisensory feedback regulates posture control and continuously updates the internal model of body's position which in turn forwards motor commands adapted to the environmental context and constraints. The peripheral localization of the vestibular system, close to the cochlea, makes vestibular damage possible following cochlear implant (CI) surgery. Impaired vestibular function in CI patients, if any, may have a strong impact on posture stability. The simple postural task of quiet standing is generally paired with cognitive activity in most day life conditions, leading therefore to competition for attentional resources in dual-tasking, and increased risk of fall particularly in patients with impaired vestibular function. This study was aimed at evaluating the effects of postlingual cochlear implantation on posture control in adult deaf patients. Possible impairment of vestibular function was assessed by comparing the postural performance of patients to that of age-matched healthy subjects during a simple postural task performed in static (stable platform) and dynamic (platform in translation) conditions, and during dual-tasking with a visual or auditory memory task. Postural tests were done in eyes open (EO) and eyes closed (EC) conditions, with the CI activated (ON) or not (OFF). Results showed that the postural performance of the CI patients strongly differed from the controls, mainly in the EC condition. The CI patients showed significantly reduced limits of stability and increased postural instability in static conditions. In dynamic conditions, they spent considerably more energy to maintain equilibrium, and their head was stabilized neither in space nor on trunk: they behaved dynamically without vision like an inverted pendulum while the controls showed a whole body rigidification strategy. Hearing (prosthesis on) as well as dual-tasking did not really improve the dynamic postural performance of the CI patients. We conclude that CI patients become strongly visual dependent mainly in challenging postural conditions, a result they have to be awarded of particularly when getting older.

## Introduction

The peripheral vestibular system is made of angular (semi-circular canals) and linear (otoliths) sensors providing the brain with sensory signals about three-dimensional head rotations and translations. The vestibular signals project to the vestibular nuclei, which in turn project to the spinal cord, the cerebellum, the thalamus, the parieto-insular vestibular cortex and related cortical areas processing the vestibular input. These descending and ascending pathways are known for their functional role in posture control and equilibrium function, gaze stabilization, self-motion perception, and spatial navigation (see Lopez and Blanke, 2011, for review). Location of the peripheral vestibular system in the inner ear, close to the cochlea, makes vestibular damages possible following cochlear implant (CI) surgery, impairing posture control and the patients' quality of life.

The literature on CI surgery and vestibular function is rather conflicting. No significant adverse effects were observed using behavioral tests (walk across and tandem tests), clinical vestibular examination (caloric and rotational chair tests), or subjective evaluation with the dizziness handicap inventory (Kluenter et al., 2009). Very uncommon loss of vestibular function (one patient among eleven) was shown with the head impulse test, a physiologically relevant stimulation able to detect subtle changes in the functioning of individual semicircular canals (Migliaccio et al., 2005). Significant improvement of both static (Kluenter et al., 2010) and dynamic (Buchman et al., 2004) balance were even observed in CI patients, and significant increase in vestibular responsiveness was noted also in CI patients during air-caloric stimulation (Ribári et al., 1999).

A retrospective case review indicates, however, that approximately three-quarters of adults with implants had experienced vertigo and imbalance (Steenerson et al., 2001). The risk of vestibular function loss depends on the CI surgical technique (Todt et al., 2008), and the vestibular deficits *per se* are function of the evaluation method (subjective questionnaires vs. objective measurements of ocular motor or postural responses). Increased postural instability has been attributed to undesirable vestibular system stimulation by the auditory electrical prosthesis (Black et al., 1978). Electrical current spread from the implant device to the vestibular nerve was suggested also by Ito (1998), who reported 18% of CI patients with dizziness when they used their implant device. The risk of damaged vestibular function in CI patients has been estimated to one third (Huygen et al., 1995) or more (Klenzner et al., 2004), up to two thirds (Van den Broek et al., 1993) with the caloric and velocity step tests. A postoperative peripheral vestibular deficit was reported in 40% of CI patients tested with electronystagmography, computerized dynamic posturography and harmonic acceleration testing (Brey et al., 1995). The authors showed a significant drop of the caloric response of the implanted ear for the older patients (over 60 years of age; *N* = 10), no change for the younger group (under 60 years of age; *N* = 7). Postoperatively, 67% of the older patients had positional nystagmus with EC while 30% was found in the younger. In the CI older patients, balance complaints and vestibular rehabilitation were observed more frequently. The older ears should be more prone to permanent injury after CI surgery (Enticott et al., 2006). Vestibular disturbances were attributed to transient canal function impairment in 20% of cases (Vibert et al., 2001).

Although most of these studies were performed during the acute stage of CI surgery and showed a resolution of the vestibular symptoms within days or weeks, some patients exhibited more persistent disturbances of balance. Chronic, persisting dizziness was attributed to saccular impairment (Basta et al., 2008). Histo-pathological data showed that CI surgery did not cause deafferentation at the periphery, but induced cochlear hydrops accompanied by saccular collapse responsible for attacks of vertigo of delayed onset (Handzel et al., 2006). Late-onset postural symptoms were reported by Shoman et al. (2008) less than 10% of their CI patients, and spells of vertigo occurring later than 1 month after CI surgery were observed by Kubo et al. (2001), Ito (1998) in 16 and 8% of their CI patients, respectively. Dizziness after implantation was seen in 39% of the CI patients (Fina et al., 2003), with the majority experiencing delayed, episodic onset of vertigo. These findings suggest that inner ear lesions due to CI surgery can develop gradually and lead to chronic changes in posture control.

The present study was aimed at examining the possible delayed effects of cochlear implantation on posture control in postlingual CI adult patients. The originality of this investigation was to evaluate the role of visual and auditory inputs suppression, and of a concomitant cognitive task (dual-tasking), on both static and dynamic postural performances of CI patients compared to healthy subjects.

## Materials and methods

### Subjects

Thirteen healthy subjects (*M*_age_ = 39.5 years, *SD*_age_ = 9.1; 6 males and 7 females) and 16 patients with unilateral CI (*M*_age_ = 59.7 years, *SD*_age_ = 12.3; 10 males and 6 females) participated in the experiment. The two groups did not differed in terms of height (*M*_height_ = 172.7 cm, *SD*_height_ = 9.5 for the controls, *M*_height_ = 167.9 cm, *SD*_height_ = 8.3 for the patients), and weight (*M*_weight_ = 65.7 kg, *SD*_weight_ = 13.1 for the controls, *M*_weight_ = 72.6 kg, *SD*_weight_ = 13.6 for the patients). All subjects provided informed consent before their participation. The experimental protocol was approved by the local Ethics Committee (CCPPRB, 2010: Université de Provence) and followed the recommendations of the Helsinki declaration.

All healthy subjects were included on the basis of the following criteria: no previous physical, neurological, or sensory disorders, no medication that might influence their balance or their cognitive performance, no history of falls in the previous 12 months, no postural and gait disorders and no vestibular deficit.

Cochlear implanted (CI) patients had received unilateral cochlear implantation 1–6 years before their participation to the present study. Nine patients had been implanted on the right side and 7 on the left. All cochlear implantations had been performed by Dr Renaud Meller. All patients suffered from deep unilateral deafness (≥90 Db) and 13 among the 16 patients had deep hearing loss on the non-implanted ear also. The mean percentage of hearing loss was 90% on the implantation side, and 5 subjects wore a hearing aid on the controlateral ear. Origin of deafness was either congenital (2 subjects), brutal (6 subjects), or progressive (8 subjects). Almost all patients (14/16) had participated to a post-operative rehabilitation (lip reading: Table 1). The background of the CI patients regarding their vestibular status has not been controlled. Only oto-neurological examination (performed before CI surgery) and a questionnaire (done at the moment of the postural investigation) were available. Oto-neurological examination had revealed no vestibular loss with the caloric test. Data from the questionnaire appreciating the possible involvement of CI surgery-induced vestibular damages on the long term showed that few of the CI patients complained of vertigo and postural instability with or without vision, during head movements, in supermarkets (3/16), and with their prosthesis off (2/16). Most of the CI patients reported not being subject to motion sickness (14/16) (Table 1).

**Table 1:** **Questionnaire on the long term self evaluation of the effects of CI surgery on the day life activity of the CI patients**.

**Vertigo**	**Instability**					**Motion sickness**	**Lip reading**
	**Light**	**Darkness**	**Head motion**	**Prosthesis off**	**Supermarket**		
3/16	3/16	3/16	3/16	2/16	3/16	2/16	14/16

The fractions in each column indicates the number of patients among the total population (N = 16) reporting vertigo, instability or motion sickness, and the number of patients submitted to lip reading rehabilitation after CI surgery.

### Experimental procedure

#### Evaluation of the postural performance: posturography

All participants were asked to stand quietly on a static/translational platform (Synapsys, Marseille, France) described in a previous paper (Ghulyan et al., 2005), with the feet aligned to the vertical projection of their shoulders. Subjects were tested in static (fixated support) and dynamic [translation in the anteroposterior (AP) direction] conditions, with their eyes open (EO) or their EC, with hearing (prosthesis ON) or without hearing (prosthesis OFF in patients and white noise inside a helmet in healthy subjects), with a concurrent auditory Spatial memory Task (audi ST, prosthesis ON) or visual Spatial memory Task (visu ST, prosthesis OFF). The participants did not wear their shoes during the postural tasks.

***Static condition.*** Recordings of 25 s duration were first performed in the light (EO) while the subjects fixated a visual target placed 2.5 m in front of them, at eye level. In the EC condition, they were asked to look at the memorized visual target. In both situations, they were instructed to maintain their balance on the static platform. The limits of stability (prosthesis ON) were measured in EO and EC conditions by asking the subjects to lean as far as possible, circularly, in all directions. They were instructed to lean their whole body without moving the feet, first forward, then to the right, backward, to the left, and again forward, at their own velocity.

***Dynamic condition.*** Recordings of 25 s duration were then used in the dynamic condition. Subjects were asked to keep their balance and to avoid stepping on the platform which moved fore and aft sinusoidally at the 0.5 Hz frequency. The amplitude of the platform translation was 7 cm. The 0.5 Hz sinusoidal translation frequency was chosen on the basis of our previous studies that showed it was both a challenging postural task and a dynamic postural perturbation which did not induce falls in aged healthy subjects (Bernard-Demanze et al., 2009) as well as in compensated unilateral vestibular loss patients (Young et al., 2012). Two experimenters stood just behind the patients to prevent falling in case of equilibrium loss. The prosthesis ON condition always preceded prosthesis OFF.

The postural tests have been done first in the static condition, and then in the dynamic condition. This non-random order was chosen to allow patients to perform all tests without stress, familiarizing them with easier tests before the more challenging tests.

#### Cognitive tasks

In the cognitive spatial task, subjects performed a multi-step translation on a 3 × 3 cell imaginary grid. From a starting location at the center of the grid, subjects were instructed to move mentally by following the auditory instructions or the visual instructions projected on a screen in front of them (move step by step to the right, to the left, backward, and forward), and to remember their new localization on the grid (cf. Bernard-Demanze et al., 2009). The cognitive task was performed without verbal expression to exclude destabilizing effects related to articulatory processes (Yardley et al., 2001; Dault et al., 2003). At the end of the exercise, subjects were asked to give their answer, but the experimenter provided no indication on the nature (right or wrong) of their performance.

#### Cognitive pre-tests

Cognitive pre-tests were aimed at evaluating the capacity of the subjects to perform the spatial task and to determine the time-interval between two instructions allowing them to correctly perform each task. During the cognitive pre-tests, the subjects were seated in front of a computer keyboard, and visual or auditory instructions were delivered on the screen or through the computer loudspeakers, respectively. The first instruction was given 5 s after the initiation of the trial. After each instruction, the subjects responded by pressing a button on the keyboard, which triggered the subsequent auditory or visual instruction. A sound indicated the end of the trial.

For each cognitive task (auditory and visual), six trials made up of 20 instructions were performed and each instruction was presented for 500 ms. Each subject's mean inter-stimulus-interval (mean _ISI_) was calculated on the basis of the number of trials (*n*) performed for each cognitive task according to the formula: mean _ISI_ = ∑ [T − (20 × 500)/20]_*n*_ where:

- *T* was the total duration of the trial, in seconds,
- 20 × 500 ms the total presentation time of the 20 instructions in the trial,
- *n* the number of trials.

The mean _ISI_ reflected the baseline cognitive abilities of each subject and was taken as the subject's own reference. For both tasks, the subjects had to report the result of their auditory and visual spatial memory calculations at the end of the experimental trial, and the trial was excluded if the result was false. No information regarding scores was provided between trials.

The mean number of trials with correct results was 6/6 in the controls, 5/6 in the CI patients for both auditory and visual spatial tasks. As a rule, CI patients had a significantly increased mean _ISI_ compared to healthy subjects for the auditory ST condition (*p* < 0.001) but not for the visual ST condition (*p* > 0.05).

***Cognitive test.*** During dual-tasking, successive instructions were presented automatically at each subject's own measured mean _ISI_ by condition (audi ST or visu ST). Since the recording time remained constant for all participants, the number of instructions per trial varied from one subject to another, and as such the CI patients received three times the mean number of instructions per trial (*p* < 0.0001) for the auditory ST condition (*N* = 37 vs. 12) and the same number (*p* > 0.05) for the visual ST condition (*N* = 11) compared to the healthy participants. In this way, the cognitive load was as similar as reasonably achievable between groups and between subjects, and this allowed a more valid inter-group comparison during dual-tasking.

#### Evaluation of the postural performance: motion analysis

Head and body motion recordings were made along with the postural recordings of the Center of Pressure (CoP) displacements. The head and body positions and their stabilization in space were recorded using a motion analysis system (Codamotion, Charnwood Dynamics, UK) sampled at 100 Hz. Two active markers were located in the infra-orbital and acoustic meatus on one side of the face to denote the Frankfurt plane, and to enable accurate analysis in all three space dimensions (cf. Tardieu et al., 2009). Head angular displacement during platform translation was measured in the X-Y, X-Z, and Y-Z planes. Head position in space was defined as the average of each head angle while head stabilization was defined as the standard deviation of the head angles. The gain of head displacement was computed as the ratio of head motion in space to platform motion.

Four other optical markers were placed on the knees and hips, and two supplementary ones were located on the platform itself. Similar calculations were made to obtain the knee and hip gains. The pattern of gains across the body characterizes the posture control strategy. A gain close to unity at the knees and tending to decrease close to zero at the head indicates a strategy of head stabilization relative to space. In contrast, gains remaining close to 1 for all markers (knees, hips, and head) point to whole-body stabilization over the feet, that is, a “rigidification” strategy (see Young et al., 2012).

### Data analysis

#### Postural performance during quiet standing

Displacements of the CoP were used to measure the limits of stability and the postural performance of the subjects. This traditional approach has been complemented by a more accurate non-linear analysis of CoP displacements using the wavelet transformation. The wavelet analysis software (PosturoPro, Framiral, Cannes) provides a time-frequency chart of body sway and a 3D representation of body sway under both static and dynamic conditions (see Lacour et al., 2008). This method gives access to the changes in the frequency components of body sway with time, the third dimension calculated as the decimal logarithm of the spectral power being given on the 3D map by a color code.

Postural performance in static conditions was evaluated through a postural instability index (PII) derived from the wavelet plots (cf. Bernard-Demanze et al., 2009). The PII values were computed for three frequency bands (F1: 0.05–0.5 Hz; F2: 0.5–1.5 Hz; F3: 1.5–10 Hz) corresponding to frequency domains mostly related to vision (F1) and vestibular/somatosensory (F2) contribution to posture control. As a rule, power in the high band (F3) is not present in healthy subjects during quiet standing, but it can be seen with aging, in postural pathologies, and of course in dynamic postural conditions. The PII values were calculated from both the spectral power recorded in a given frequency range, and the total time during which the spectral power of the different body sway frequencies in this given frequency range tend to be canceled by the posture control mechanisms (see Lacour et al., 2008). Indeed, the wavelet plots showed the spectral power for a particular frequency of body sway was not constant over time, but varied and tended to come close to zero. The algorithm used to compute the PII was as follows:

PII=∑x∑y SP (F1, F2, F3)/TC (F1, F2, F3)

where SP and TC are the spectral power (in arbitrary units) and time cancellation (in seconds) for each of the three frequency bands. In healthy adults, the PII value recorded in the eye open condition during quiet standing is close to unity while it is significantly increased (up to 4–5) in pathological cases or in older adults in dual-tasking (Bernard-Demanze et al., 2009).

#### Postural reactions to linear AP translations

In dynamic condition, the spectral power density was computed for the 0.5 Hz frequency peak, corresponding to the platform translation frequency. The spectral power peak was expressed in arbitrary units, which ranged from 10^3^ to 10^9^ depending on the nature (static vs. dynamic) of the postural task.

### Statistical analysis

#### Static condition

Two parameters describing body sway during quiet standing were analyzed: the limits of stability and the PII. The limits of stability parameter was analyzed using repeated-measures analyses of variance (ANOVAs) with “group” (healthy subjects vs. CI patients) as the between-subject factor, and visual condition (EO vs. EC) as within-subject factors. The PII was analyzed using repeated-measures ANOVAs with “group” (healthy subjects vs. CI patients) as the between-subject factor, visual condition (EO vs. EC) and condition (prosthesis ON, prosthesis OFF, audi ST) as within-subject factors. This parameter was also analyzed for the two groups in EO condition with a separate ANOVA with condition (prosthesis ON vs. prosthesis OFF), and cognitive task (audi ST vs. visu ST) as within-subject factors.

#### Dynamic condition

Separate ANOVAs were applied to dynamic postural control. Postural response to platform sinusoidal translation at the 0.5 Hz stimulus frequency was evaluated by the spectral power peak provided by the wavelet analysis. The gain of the head, hip and knee was computed using measurements from the motion analysis system, by calculating the ratio of the amplitude of displacement of each marker at its specific segmental level to the amplitude of platform displacement.

Results were considered significant for *p* < 0.05.

## Results

### Postural control of the CI patients in static conditions

**Figure 1:**
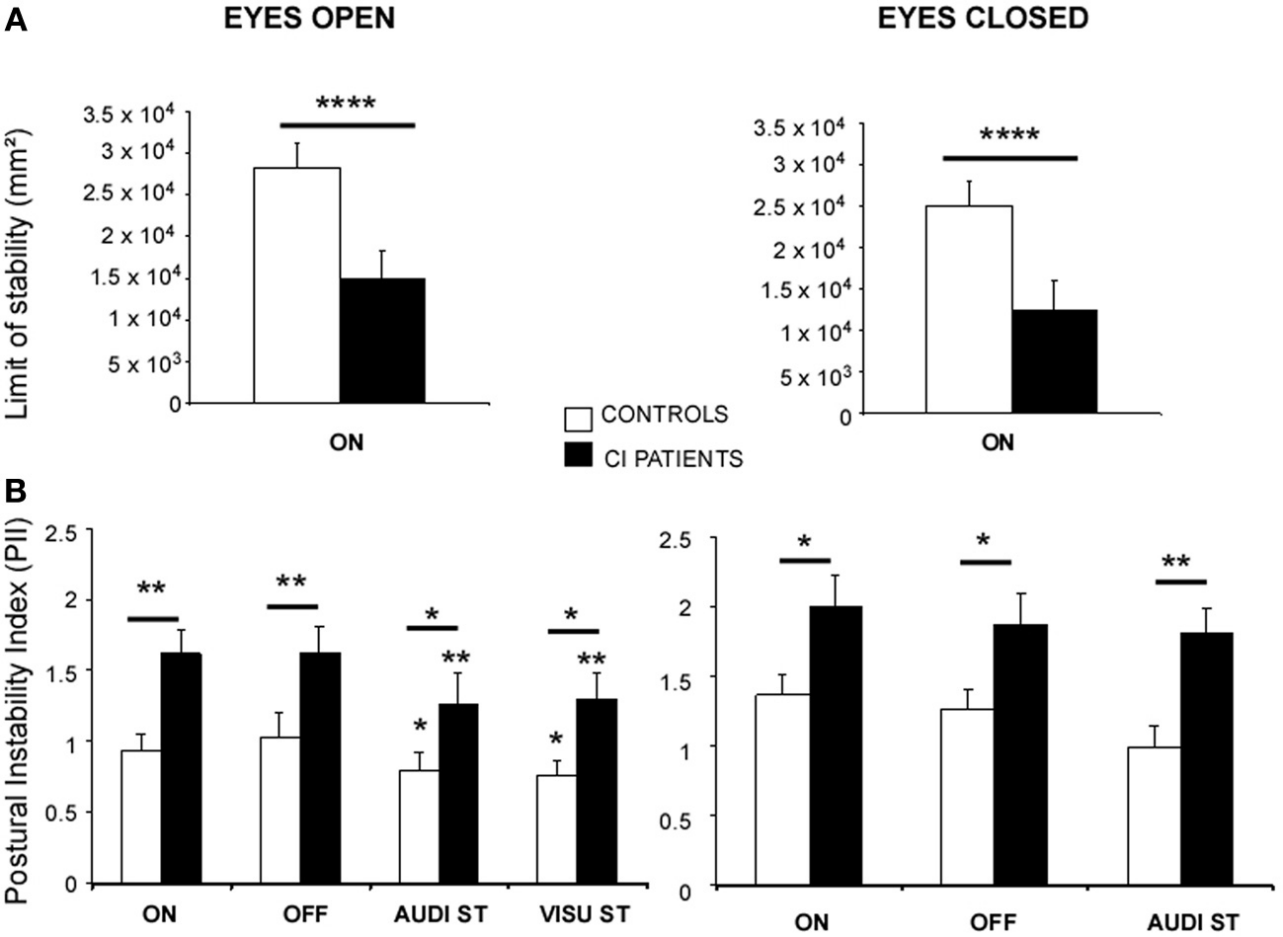
**(A,B)** Posture control of the CI patients in static conditions. **(A)** Limits of stability of the CI patients (filled histograms) compared to the controls (open histograms) in the eyes open (EO) and eyes closed (EC) conditions. The stability limits are expressed as the energy of the spectral power density recorded in the 0.05–0.5 Hz frequency range while the subjects were standing on the platform and asked to move voluntary as far as possible in the forward/backward and left/right directions, without moving the feet. ^****^Significant differences (*p* < 0.0001) between the CI patients and the controls. ON indicates that hearing is present. **(B)** Postural Instability Index (PII) calculated from the 3D posturographic map of the center of pressure recordings obtained with the wavelet analysis. Recordings were made in subjects standing quietly on the platform with (EO) or without (EC) vision, with (ON) or without (OFF) hearing, and during dual-tasking (DT) with a concomitant cognitive task consisting of a visual (visu ST) or an auditory (audi ST) memory task. The PII values are expressed on the ordinates; significant differences between the CI patients (filled histograms) and the controls (open histograms) are indicated by asterisks (^*^p < 0.05; ^**^p < 0.01).

The ANOVA performed on the limits of stability showed significant differences between the CI patients and the healthy subjects [*F*_(1, 27)_ = 34.23; *p* < 0.000001] in both EO (*p* < 0.00001) and EC (*p* < 0.00001) conditions. The Figure 1A illustrates the stability limits recorded in the CI patients (with their prosthesis ON) and in the controls in both EO and EC conditions. The histograms plot the mean energy recorded in the 0.05–0.5 Hz frequency range while they tilt voluntary their whole body forward, to the left, backward, to the right and again forward. The spectral power density recorded in this low frequency range was significantly reduced by around 50% in the CI patients, indicating that they had reduced voluntary body sways compared to the healthy subjects.

The ANOVA performed on the PII calculated from the whole 3D chart provided by the wavelet transform showed also significant differences between the two groups [*F*_(1, 27)_ = 13.56; *p* < 0.001] in all the experimental conditions tested. The Figure 1B illustrates the mean PII values recorded with and without vision (EO and EC), with and without hearing (ON and OFF conditions), and during dual-tasking with the cognitive auditory (audi ST) and visual (visu ST) memory tasks.

As a general rule, modifying the experimental conditions had similar consequences on the postural performance of both controls and CI patients. Suppression of the auditory cues had no effect at all, suppression of visual information increased the PII values (*p* < 0.05), and dual-tasking with the cognitive auditory or visual memory tasks reduced the PII values (*p* < 0.05) compared to the single quiet standing postural task. On the other hand, whatever the postural task, the PII values of the CI patients were significantly increased compared to the controls, indicating that their posture control was not as efficient as in healthy subjects. The two groups differed significantly in the EO condition with and without hearing (*p* < 0.01), and during dual-tasking with the auditory and visual memory tasks [*F*_(2, 24)_ = 3.87; *p* < 0.05; *F*_(2, 30)_ = 9.17; *p* < 0.001, for the controls and the patients, respectively]. The CI patients still differ significantly from the healthy subjects in the EC condition with and without hearing (*p* < 0.05) and during dual-tasking with the auditory memory task (*p* < 0.01).

### Postural control of the CI patients in dynamic conditions

In the dynamic condition, the support was translated sinusoidally in the antero-posterior direction at the 0.5 Hz frequency. The ANOVA performed on the spectral power density peak at this stimulus frequency showed that group (CI patients vs. controls), visual condition (EO vs. EC), hearing condition (ON vs. OFF), and dual-tasking (with the auditory memory task) constituted the main fixed effects constituting the sources of variations among the subjects.

**Figure 2:**
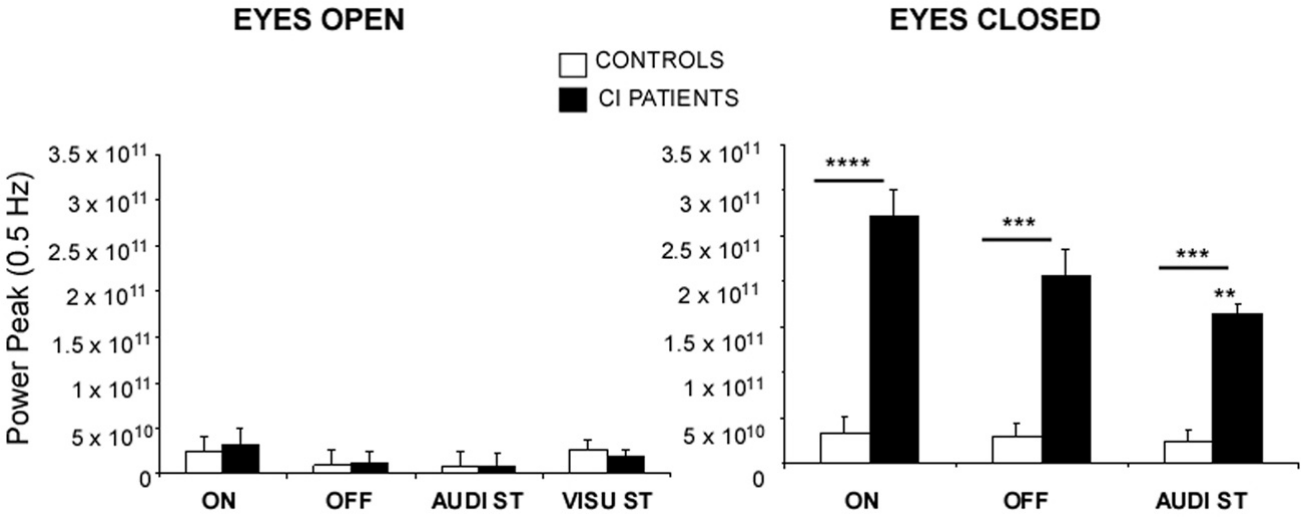
**Posture control of the CI patients in dynamic conditions**. The spectral power density peaks provide by the wavelet transform at the 0.5 Hz stimulus frequency of sinusoidal platform translation in the for-aft direction are expressed on the ordinates for each of the different experimental conditions: with (EO) or without (EC) vision, with (ON) or without (OFF) hearing, and during dual-tasking with a concomitant cognitive task consisting of a visual (visu ST) or an auditory (audi ST) memory task. Significant differences between the CI patients (filled histograms) and the controls (open histograms) are indicated by asterisks (^**^p < 0.01; ^***^p < 0.001; ^****^p < 0.0001). Note the strong impact of eye closure in the CI patients.

The Figure 2 illustrates the mean spectral power density peaks recorded in these different experimental conditions. The power peaks were not significantly different in the two groups when tested in the EO condition, whatever the experimental condition. In the EC condition, the spectral power density peaks were increased in both groups compared to the condition with vision [*F*_(1, 27)_ = 28.86; *p* < 0.0001]. But the power peaks were drastically increased in the CI patients compared to the controls [*F*_(1, 27)_ = 22.73; *p* < 0.0001] in all experimental conditions {ON vs. OFF vs. audi ST*:* [*F*_(2, 54)_ = 7.39; *p* < 0.0001]}. On the average, the peak amplitude of the spectral power density corresponding to the 0.5 Hz stimulus frequency was 3.1 × 10^10^ vs. 2.7 × 10^11^ in the hearing ON condition, 2.8 × 10^10^ vs. 2.2 × 10^11^ in the hearing OFF condition, and 2.7 × 10^10^ vs. 1.8 × 10^11^ in dual-tasking with the auditory memory task, for the controls and the CI patients, respectively. A significant interaction group × task was found in the EC condition [*F*_(2, 54)_ = 2.97; *p* < 0.05], the spectral power density peak being decreased in the CI patients during dual-tasking while it remained unchanged in the controls (*p* < 0.01).

**Figure 3:**
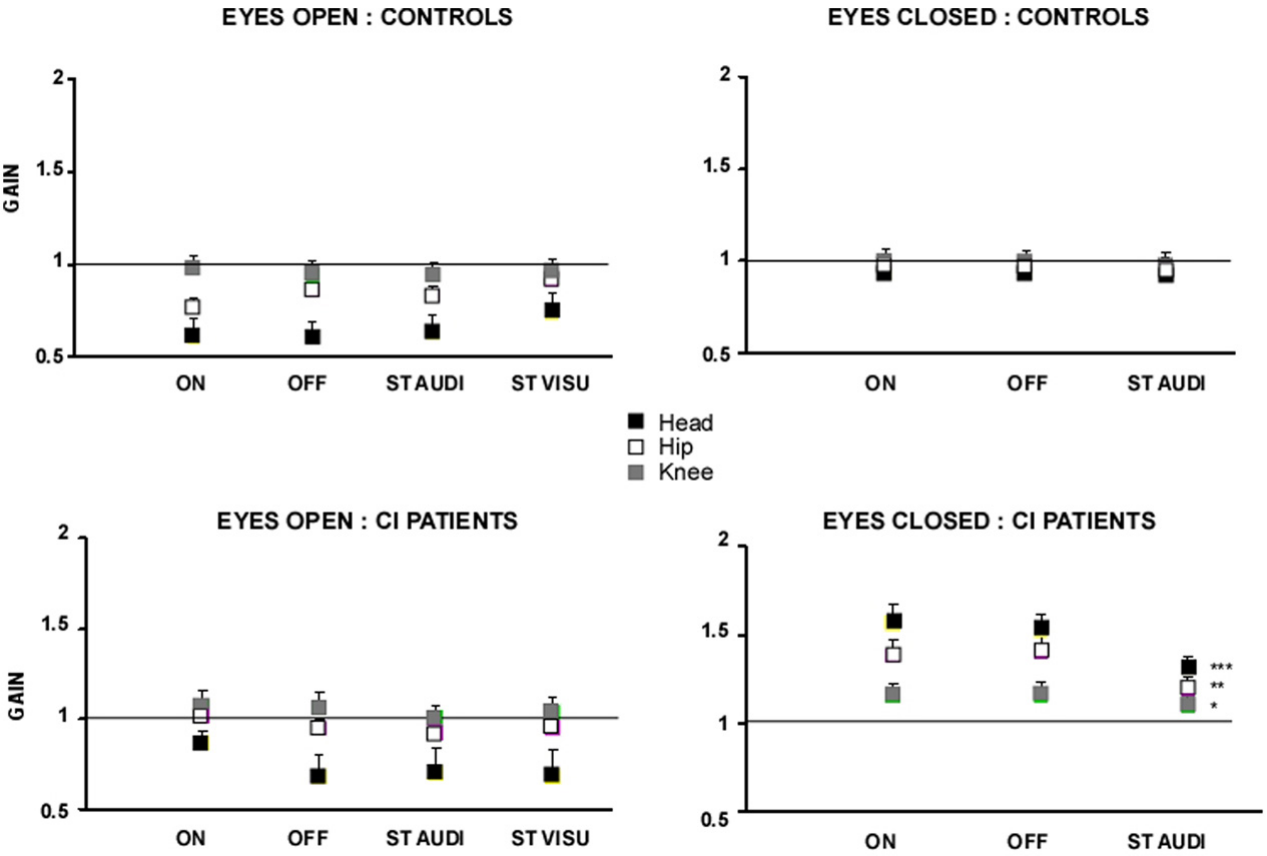
**Gain of the head, hip and knee body segments during sinusoidal platform translation at the 0.5 Hz frequency**. The gains of the head (filled black squares), hip (open squares), and knee (filled gray squares) body segments, evaluated from motion analysis (Codamotion), were calculated as the ratio between platform displacement and displacement of the different body segments. Gains close to unity mean that the body segments move in phase and with the same amplitude as the platform; lower and higher gains for the head, for instance, indicate good and poor head stabilization in space, respectively. The gain values are expressed on the ordinates for each of the experimental condition tested: with (EO) or without (EC) vision, with (ON) or without (OFF) hearing, and during dual-tasking with a concomitant cognitive task consisting of a visual (visu ST) or an auditory (audi ST) memory task. Significant differences between the experimental conditions are shown by asterisks (^*^p < 0.05; ^**^p < 0.01; ^***^p < 0.001).

The gains of the head, hip and knee displacements with respect to platform displacement are illustrated in the Figure 3 for all the experimental conditions tested. In the EO condition, the two groups behaved similarly, thus corroborating the data previously shown with the peak of spectral power density. With vision, there was a bottom–up gain decrease from foot to head: the knee gain was close to unity, indicating that the knees (and feet) follow the platform motion, while the gains of the other segments were more and more reduced as far as the head level was concerned [*F*_(2, 54)_ = 154.84; *p* < 0.0001]. These data point to a strategy of head stabilization in space during the for-aft translation of the support. Paradoxically, an interaction group × task was found in the CI patients [*F*_(3, 81)_ = 8.86; *p* < 0.0001] who showed a poorer head stabilization in space with their prosthesis ON (*p* < 0.001).

This head stabilization strategy was not preserved in both groups in the EC condition (Figure 3). The control subjects showed a gain close to unity for all body segments, in all experimental conditions, indicating that they swayed in block with the platform displacement. They exhibited a strategy of head stabilization on trunk, resulting in a rigid whole body translation in space. The CI patients showed on the other hand head stabilization neither in space nor on trunk. They behaved like an inverted pendulum with strongly increased gains at the different segmental levels that decreased in a top–down process from head to foot (gains: 1.6 ± 0.11, 1.4 ± 0.09, and 1.1 ± 0.07 for the head, hip and knee, respectively, in the prosthesis ON condition). No significant change was observed without hearing (prosthesis OFF). During dual-tasking with the auditory memory task, the different segmental gains were reduced significantly (*p* < 0.001, *p* < 0.01, and *p* < 0.05 for the head, hip, and knee, respectively) but remained higher compared to the controls (*p* < 0.001).

## Discussion

### Posture control of the CI patients in static conditions

In non-challenging postural conditions, with a stable support, the postural performance of the CI patients differed significantly from the healthy subjects. Their stability limits were reduced and their PII was increased, particularly in the EC condition. Postural performance remained unchanged with or without hearing and was similarly affected by dual-tasking in both groups.

These data clearly show that adults CI patients examined a long time period after CI surgery (1 year or more) have a less efficient posture control system than healthy controls. The difference in the mean age of the CI patients (59.7 years) and the controls (39.5 years) of the present study very unlikely plays a significant role. Indeed, the mean PII values (unpublished normative data) recorded with vision in 30–40 years old (*N* = 90; PII = 0.72 ± 0.44) and in 50–60 years old (*N* = 84; PII = 0.94 ± 0.61) healthy subjects were significantly lower than in our CI patients (PII = 1.6 ± 0.35; *p* < 0.001). The differences were still significantly different without vision (PII = 0.93 ± 0.63, 1.13 ± 0.58, and 2.0 ± 0.46 in the 30–40, 50–60 years old controls and CI patients, respectively). In addition, no significant differences were observed between younger and older healthy adults on the postural task performance (mean PII value and spectral power density) recorded during quiet standing (Bernard-Demanze et al., 2009).

Because of the retrospective nature of our investigation, the pre-operative vestibular function of the CI patients was not available, and in addition their hearing level was not uniforme. For the same reason, the postural tests have not been done before CI surgery, so that it was not possible to compare the postural data before and after CI surgery. These are the main limitations of the present study. In spite of the limited number of included CI patients, significant differences compared with healthy subjects were found, however, for most of the postural tests we have performed. Our results could support previous investigations reporting delayed adverse effects of CI surgery, with occurrence of postural symptoms (Shoman et al., 2008), dizziness (Fina et al., 2003), vertigo spells (Ito, 1998; Kubo et al., 2001), and drop in the caloric response (Brey et al., 1995). Impairment of saccular/utricular function has been reported also (Basta et al., 2008). The patients' background was not controlled, as said just before, and any hypothesis on a link between CI surgery and vestibular function impairment would be purely speculative. But data from the subjective questionnaire showed they pursued normally their professional activities, and most of them had no complaints that could be related to vestibular dysfunctions: vertigo spells and instability during head motion were very uncommon complaints (3/16). And if CI vestibular damages had occurred after CI surgery, they looked like very well compensated over time. In fact, some similarities can be found between our CI patients and the compensated Menière's disease patients we have tested in a previous study (the patients had undergone a surgical vestibular neurotomy on their affected side 2–4 years before: cf. Young et al., 2012). The vestibular loss patients as well as the CI patients relied more on vision and spent more energy maintaining balance than controls. These data corroborate also a recent study showing that equilibrium function without vision was lowered in CI patients (Kluenter et al., 2010). The anxiety level could also modify the postural performance as demonstrated in both healthy and pathological subjects (Young et al., 2012). In this latter study, the Short Anxiety Screening Test (SAST) revealed that compensated Menière's patients were more anxious than healthy controls. An increased anxiety level seems another common factor to CI patients and compensated Menière's patients, since many of our CI patients reported to feel dizzy when tired or walking slowly, to have difficulties when listening music, and to be anxious when phoning with non-familiar people. In addition, the CI patients could be anxious very likely due to their poorer postural control. It should be interesting to control the anxiety level in further investigations with CI patients. Anxiety related to greater fear of fall might be involved too. Anxiety can shorten the postural reflex pathways and lead to a kind of “rigidification” (see below, the postural strategies in dynamic condition). In order to avoid a potentially dangerous situation, we found that recovered vestibular neurotomized patients (Young et al., 2012) and our investigated CI patients reduced their voluntary stability limits, and stand more rigidly.

Suppression of hearing had no effect in both populations of CI patients and controls, indicating that auditory cues do not play a significant sensory substitution role in posture recovery, contrary to the visual cues. On the other hand, the poorer postural performance of the CI patients cannot be attributed to undesirable vestibular stimulation by the prosthesis. Interestingly, dual-tasking induced similar changes of the postural performance in both groups. When a concomitant cognitive task was present, either an auditory or a visual memory task, the postural performance was improved. This confirms a previous study we had performed in adult healthy subjects during dual-tasking (Bernard-Demanze et al., 2009). Postural performance improvement during dual-tasking can be seen as a shift of attention away from the postural task. Focusing attention on cognitive tasks delegates the postural control system to highly automatic processes, as previous reported (Huxhold et al., 2006). Following Baltes' model of task prioritization (Baltes and Baltes, 1990), we have proposed a “cognitive first principle” (Bernard-Demanze et al., 2009) for healthy younger adults during dual-tasking, that is, the mirror image of the “posture first principle” described by Shumway-Cook et al. (1997) in older subjects under dual-tasking situations. Due to the unchallenging postural context, allocating all attention resources to cognitive activity seems an optimal strategy (very likely unconscious) that does not cause resource competition and related detrimental effects. The constrained-action model (Wulf et al., 2001) that predicts interference on automatically self-organized postural behavior when attention is focused on it, has been verified: explicit instructions to focus attention on the postural task induce an increase in body sway (Vuillerme and Nafati, 2005). The CI patients used therefore the same strategy than the controls during dual-tasking, a result indicating that the cognitive functions controlling the posture control system are not modified in CI patients.

### Posture control of the CI patients in dynamic conditions

In more challenging conditions, with sinusoidal for-aft displacement of the support, both groups behaved similarly when visual information was available. They showed a strategy of head stabilization in space, the so-called stable-platform strategy proposed by Horak and Nashner (1986) in healthy subjects, and reported more recently in compensated unilateral vestibular loss patients (Young et al., 2012). Dual-tasking and suppression of hearing did not modify this strategy.

By contrast, suppression of the visual input led to different effects on the postural performance, depending on the groups. While the healthy controls performance was not affected by eye closure, the CI patients relied again more on vision, and spent considerably more energy maintaining balance with the eyes closed (EC), as shown by the strongly increased spectral power density peak recorded at the 0.50 Hz stimulus frequency of platform translation. These data collected in more challenging conditions confirm the crucial role of vision described before in the CI patients under quiet standing. Prosthesis off had no significant effects on the dynamic postural performance of the CI patients, while dual-tasking reduced significantly the spectral power density peak (1.6 × 10^11^) compared with the single postural task (2.7 × 10^11^).

More interesting are the different strategies of posture control observed when the visual cues were suppressed. Control subjects selected a head on trunk strategy, the so-called strap-down strategy described by Horak and Nashner (1986) and characterized by a rigid whole body in space. The gain at the three segmental levels tested (head, hip, and knee) was close to unity. This “stick” behavior had already been described in adult healthy subjects under increased postural threat (Young et al., 2012), in older healthy subjects in challenging postural environments (Brown et al., 2002), and in unilateral vestibular loss patients (Young et al., 2012). The CI patients showed very poor body stabilization in space. Their head was stabilized neither in space nor on trunk. They behaved like an inverted pendulum, with the feet and knee following platform displacement (gain close to 1), and with much more high gains for the hip (gain = 1.4) and the head (gain up to 1.6). This behavior is not adapted to equilibrium maintenance, and should induce falling or stepping at higher translation frequencies. Suppression of the auditory cues did not modify this behavior. The dual-tasking condition, however, reduced the gain at the three segmental levels, suggesting again a shift of the attention resources on the cognitive task (see Lindenberger et al., 2000; Li et al., 2001), and modified posture balance, with a more rigid whole body in space.

## Conclusion

The present study clearly shows that the CI patients' postural performance is lower compared to control subjects in both static and dynamic conditions, particularly without vision. They need visual inputs to control their posture and equilibrium in both quiet standing and more challenging postural conditions. That CI patients showed impaired postural performance and relied exclusively on a visual sensory substitution strategy are the most important results of this study. Visual sensory substitution is a common process described in functional recovery after stroke (Pérennou, 2006), Parkinson disease (Azulay et al., 2002) and vestibular pathology (Lacour, 2006). However, vicariant idiosyncratic processes are generally observed in vestibular loss patients. In a previous study performed in Menière's patients submitted to a curative unilateral vestibular neurotomy, we had described two different sensory substitution strategies, with half of the population (25 patients) relying mostly on vision and the other half (25 patients) relying mostly on somatosensory inputs (Lacour et al., 1997). In this paper, we had recommended different rehabilitation programs and exercises, depending on the sensory strategies used by the patients. In our population of CI patients, only the visual strategy was found. This shift toward a total visual dependency is very likely due to the rehabilitation procedure they were submitted to after CI surgery, that is, lip reading. And a too strong visual dependency may have deleterious effects on posture control (Bronstein, 1995). Specific rehabilitation programs should be proposed to CI patients, with exercises focusing more on proprioception and somatosensory cues.

We conclude therefore that chronic (>1 year) CI patients (1) have impaired postural performance, and (2) exhibit a sensory substitution strategy based on vision. The CI patients have to be aware of their visual dependency, particularly when they get older and have impairment of vision.

### Conflict of interest statement

The authors declare that the research was conducted in the absence of any commercial or financial relationships that could be construed as a potential conflict of interest.
